# Cucurbituril—assisted sensitive fluorescence detection and quantitation of naproxen drug in wastewater samples: Guest-host characterization and HPLC investigation

**DOI:** 10.3389/fchem.2022.1093231

**Published:** 2022-12-02

**Authors:** Mohammed A. Meetani, Ahmad Alhalabi, Mohammed K. Al-Tabaji, Abdulla Al-Hemyari, Haythem A. Saadeh, Na’il Saleh

**Affiliations:** ^1^ Chemistry Department, College of Science, United Arab Emirates University, Al-Ain, United Arab Emirates; ^2^ Chemistry Department, Faculty of Science, University of Jordan, Amman, Jordan

**Keywords:** naproxen, cucurbituril, fluorescence, guest-host interaction, wastewater

## Abstract

Sensitive spectrofluorometric and liquid chromatography with fluorescence detection methods have been developed for detection and determination of naproxen drug in the presence of cucurbit7uril (CB7). Fluorescence signals have been improved with the addition of CB7 to the drug aqueous solution. Fluorescence spectroscopy, mass spectrometry, ^1^H-NMR, and liquid chromatography with fluorescence detection were used to investigate the guest-host interaction of naproxen drug and cucurbiturils. Naproxen was found to form a supramolecular complex with CB7 that had a high formation constant. The optimal conditions for the interaction were discovered using spectroflurometry to be 0.2 mg/ml of CB7, 2.4 μg/ml of naproxen drug, and pH10. A 1:1 complex between naproxen and CB7 is revealed by proton NMR and tandem mass spectrometry. Using the standard addition calibration method, an HPLC with a fluorescence detector was used to detect naproxen in influent and effluent wastewater samples. Finally, it was discovered that the measured concentrations of naproxen in the influent and the effluent wastewater were 1.87 × 10^−4^ ppb and 2.1 × 10^−5^ ppb, respectively. This was done by sample enrichment, which reduced the 1000 mL into 1 ml.

## 1 Introduction

Nonsteroidal anti-inflammatory medicines (NSAID), known as profens, are widely prescribed throughout the world. Profens such as ibuprofen, naproxen, flurbiprofen, suprofen, and Ketoprofen are frequently detected at ng/L in both treated wastewater and surface water due to poor removal rates during wastewater treatment (Ali et al., 2017). Naproxen has been found in a variety of water sources, including treated waste water, drinking water, and groundwater (Madikizela and Chimuka, 2017b; Madikizela et al., 2017). The concentrations that have been observed ranged from ng/L to μg/L. These concentrations, although low, may have a negative effect of long-term exposure on nontarget organisms, especially when naproxen is mixed with other drugs (Wojcieszyńska & Guzik, 2020).

Naproxen, also known as (+)-(S)-2-(6-methoxynaphthalen-2-yl) propanoic acid, is a pain reliever that is also used to treat inflammatory diseases, gout, and fever (Zhang et al., 2019; Han & Küçükgüzel Ş, 2020). It has been extensively studied in pharmaceutical research, including bioavailability (Wang-Smith et al., 2012), biotransformation (Sugár et al., 2019), and formulations (García-Herrero et al., 2019). It has been detected in plasma (Landry et al., 2015), urine (Dionísio et al., 2020), and saliva (Cicolani et al., 2021) using a variety of analytical techniques such as liquid chromatography with UV-Vis, fluorescence, mass spectrometry, and electrochemistry detection (García-Herrero et al., 2019; Dionísio et al., 2020).

It has been previously demonstrated that cucurbiturils (CBs) increase the fluorescence emission of a number of hydrophobic fluorophores (Wagner & McManus, 2003; Baglole et al., 2005; Cicolani et al., 2021). CBs are macrocyclic molecules made up of glycoluril monomers linked by methylene bridges. They are classified based on the number of glycoluril subunits present, such as CB5, CB6, CB7, CB8, CB10, and CB14. The CBs have a cavity (pore) that can accept small molecules as “guests,” forming inclusion complexes through hydrophobic or ion dipole interactions (Kim et al., 2007). The size of the pore and the environment within it can be modulated through changes to the subunits, with cavity diameters of 5.8 A°, 7.3 A°, and 8.8 Å for the CB6, CB7, and CB8, respectively, and annular depths of 9.1 Å (Kim, 2002; Jason Lagona et al., 2005). As a potential mechanism for fluorescence enhancement when the fluorophore interacts with the cucurbituril, confinement or polarity effects are primarily responsible for the noncovalent interaction (Saleh et al., 2011; Saleh et al., 2014; Alremeithi et al., 2016). As a result, when inclusion complexes form, the fluorophores’ weak fluorescence emission is altered, and it usually becomes stronger (Baglole et al., 2005; Li et al., 2010; Dong et al., 2011).

High performance liquid chromatography is an important tool for identifying and quantifying pollutants in the environment (Han et al., 2017; Basheer et al., 2020). A method for simultaneously determining ibuprofen and 17 related compounds using reversed-phase high-performance liquid chromatography (RP-HPLC) was developed and validated. This method was used to analyze seven batches of ibuprofen drug products from various manufacturers in order to control the quality of ibuprofen-containing substances (Han et al., 2017).

Several methods have been used to determine naproxen in wastewater samples. For instance, a fluorescent sensor based on molecularly imprinted carbon dots (CDs) has been developed by Li and others for the extremely selective detection of trace amounts of naproxen. A detection limit of 0.03 μM (∼7 ppb) and a linear range of 0.05–4 μM for detecting naproxen in aqueous solution were obtained (Li et al., 2021). In another case, naproxen was detected in river water and wastewater using molecularly imprinted solid-phase extraction followed by High Performance Liquid Chromatography-Photodiode Array Detection. The detection limits for naproxen in wastewater treatment plant effluent was 0.23 ppb (Madikizela et al., 2017).

Finally, HPLC with fluorescence detection in the presence of β-cyclodexrin was used for the sensitive detection and quantitation of several drugs in complex matrices (Meetani et al., 2019; Alremeithi et al., 2016; Saleh et al., 2014; Saleh et al., 2011). For instance, Alremaithi et al. have reported the determination of p-amino hipparic acid in urine samples using HPLC- FLD; β-cyclodexrin was used as a mobile phase additive to enhance the biomarker fluorescence detection through supramolecular interaction (Alremeithi et al., 2016).

There is no evidence that naproxen (guest) and cucurbiturils (host) interact supramolecularly. Despite the fact may studies have found naproxen in wastewater (Durán-Álvarez et al., 2015; Madikizela & Chimuka, 2017a). However, there were no reports of fluorescence-based naproxen sensing in wastewater using a supramolecular approach in the literature. As a result, in this study, we used fluorescence spectroscopy, ^1^H-NMR, electrospray mass spectrometry (ESI-MS), and liquid chromatography with fluorescence detection (HPLC-FLD) to investigate the interaction of naproxen drug with CB7. In addition, the drug naproxen’s quantitative determination in influent and effluent wastewater samples was assessed after solid phase extraction and concentration enrichment of the wastewater samples. CB7 was used as an additive in the mobile phase of the HPLC-FLD, taking advantage of its special hosting properties of a high binding constant.

## 2 Experimental

Naproxen, CB6, CB7, CB8, ethanol, acetone, and chloroform (HPLC grade) were provided by Sigma-Aldrich, United States . Stock and working solutions were made using double-distilled water that was acquired from the Milli-Q gradient system (Millipore). The Agilent Cary Eclipse fluorescence spectrofluorometer (Agilent, United States) and AnalytikJena SPCORD^®^ 210 spectrophotometer (AnalytikJena, Germany) were used to measure fluorescence and UV-visible absorption, respectively. Agilent 1200 LC system with fluorescence detector (FLD) was used for HPLC analysis (Agilent, United States). Liquid chromatographic separations with isocratic elution were carried out at a column temperature of 55°C using a Symmetry C18 column (150 mm, 4.6 mm, 5 m) (Waters, UK). The injection volume was 10 μL. A combination of 15:85 ethanol and 20 mM aqueous phosphate buffer made up mobile phase at pH 8.3. The final concentration of CB7 added to the HPLC mobile phase was 10 μM. The flow rate of the mobile phase was 0.8 ml/min. The fluorescence detection wavelengths were set at λ_ex_ = 232 nm and λ_em_ = 355 nm.

UV-visible spectroscopy was used to establish the naproxen ideal excitation wave length for fluorescence studies. A stock solution of 1 mM naproxen was produced in deionized water and stored in dark vial in the refrigerator, on daily basis, the experimental samples (working) solutions were prepared fresh from stock solution. CB7 solid powder (1 mg) was dissolved in a 5.0 ml of the naproxen working solution prior to the fluorescence measurement. The CB7 concentration was fixed at 1.0 mg/5 ml (0.172 mM) for fluorescence measurements, CB8 concentration was fixed at 1.0 mg/5 ml (0.150 mM), and naproxen concentration was varied. To achieve the best detection conditions, the pH was altered by adding aliquot amounts of HCl or NaOH solutions and then pH was measured using a WTW 330i pH meter with SenTix Micro glass electrode.

The Varian 400 MHz instrument was used to perform proton NMR measurements.

^1^H-NMR spectra were collected for the CB7, CB8 and naproxen separately in D_2_O solvent. Afterword, a mixture of 1.50 mM naproxen, and 0.306–2.66 mM CB7 or 1.50 mM naproxen and 0.12 mM–1.7 mM CB8 in D_2_O were measured after each incremental addition of CBs and the solution pH was set to pD 5.8.

Direct infusion into a Thermo Finnigan LTQ linear ion trap mass spectrometer (Thermo Scientific, United States) fitted with an electrospray ionization (ESI) probe was used to perform mass spectrometric observations. The mass spectrometer settings were as follow: The spray voltage was 4.5 KV, and the ion polarity was positive. The sheath gas pressure was 40 units, the spray gas was nitrogen, and the auxiliary pressure was 10 units. The collision gas was helium, and the temperature of the ion transfer tube was 300°C.

Wastewater samples were analyzed for naproxen drug using an automated extraction equipment from Horizon Technology SPE-DEX 4790, (Salem, United States), using previously reported extraction steps (Meetani et al., 2019; Maraqa et al., 2020). The Atlantic^®^ HLB-M disks with N-vinylpyrrolidone and divinylbenzene sorbents were used. A 1000 ml wastewater samples were used for the SPE, and naproxen was extracted into 20 ml of concentrated acetone: dichloromethane organic extracts before being dried with nitrogen gas flowing over them.

The sample extract was reconstituted in 1.0 ml of water: ethanol solution then it was divided equally into five small vials, each containing 0.2 ml. These five vials were spiked with a standard solution of naproxen, 100 ppb, in varying quantities (0.0, 0.1, 0.2, 0.3, and 0.4 ml). After that, each vial’s total capacity was filled to 1.0 ml with the ethanol: water solution. Afterward, samples were checked using the HPLC-FLD device.

## 3 Results

**FIGURE 1:**
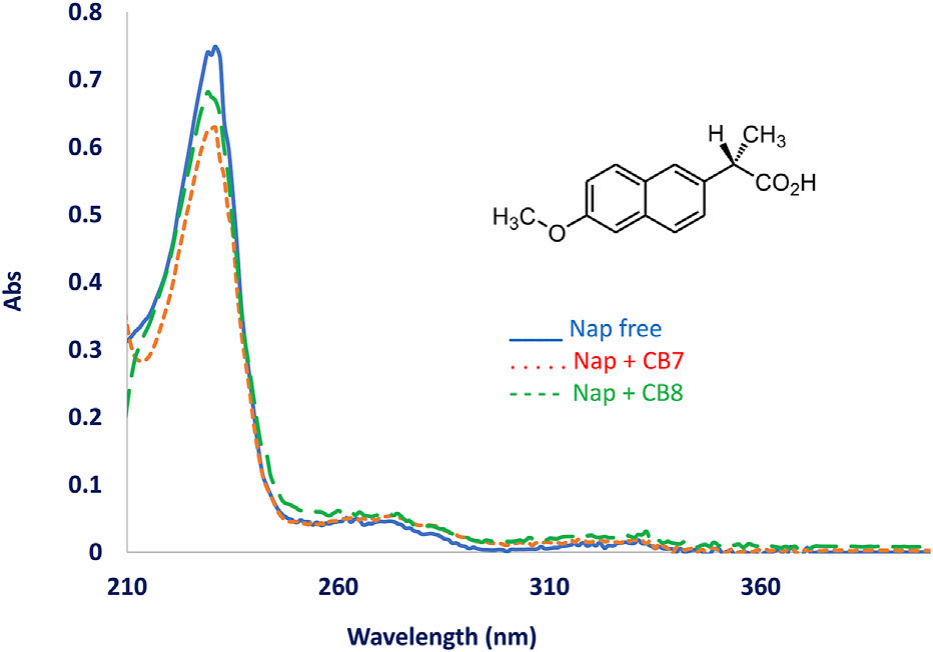
UV-Vis spectra of naproxen in aqueous solution without adding CBs (Blue solid line) and after adding CB[8] (dotted line) and CB[7]. Naproxen structure is shown at the figure top.

The UV-Visible spectra of naproxen alone and in combination with CB7 or CB8 show a small change in absorbance intensity of naproxen, as shown in Figure 1. Additionally, the height of the absorption peak was lowered and an isosbestic point was created when CB7 or CB8 were added to the drug solution. Naproxen is an excellent fluorophore, producing a bright fluorescence signal at 355 nm even at low concentrations of 10^−8^ M.

**FIGURE 2:**
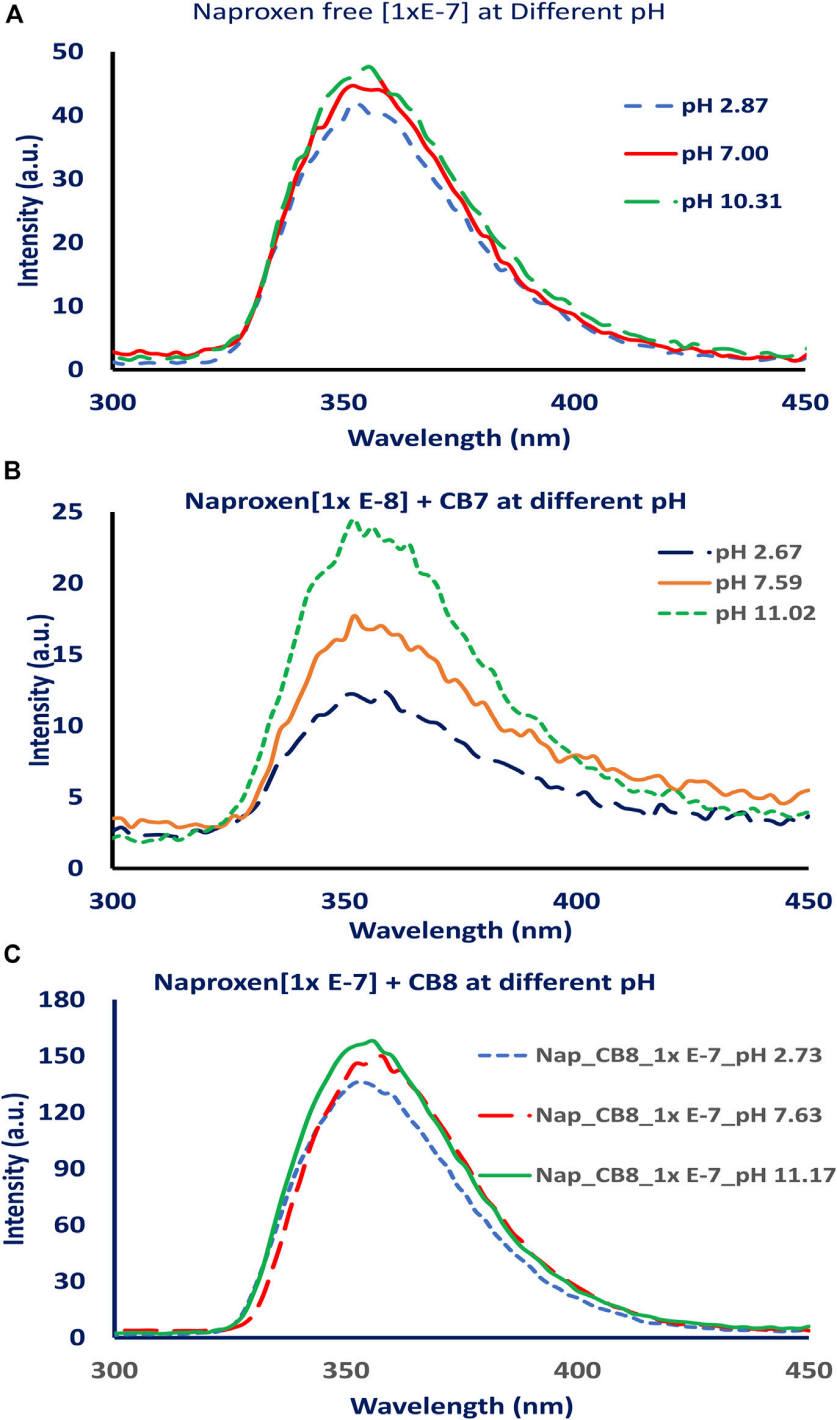
Fluorescence spectra of naproxen at different pH values (pH 2, 7 and 11), **(A)** without adding CBs, **(B)** after adding CB7, and **(C)** after adding CB8.

**FIGURE 3:**
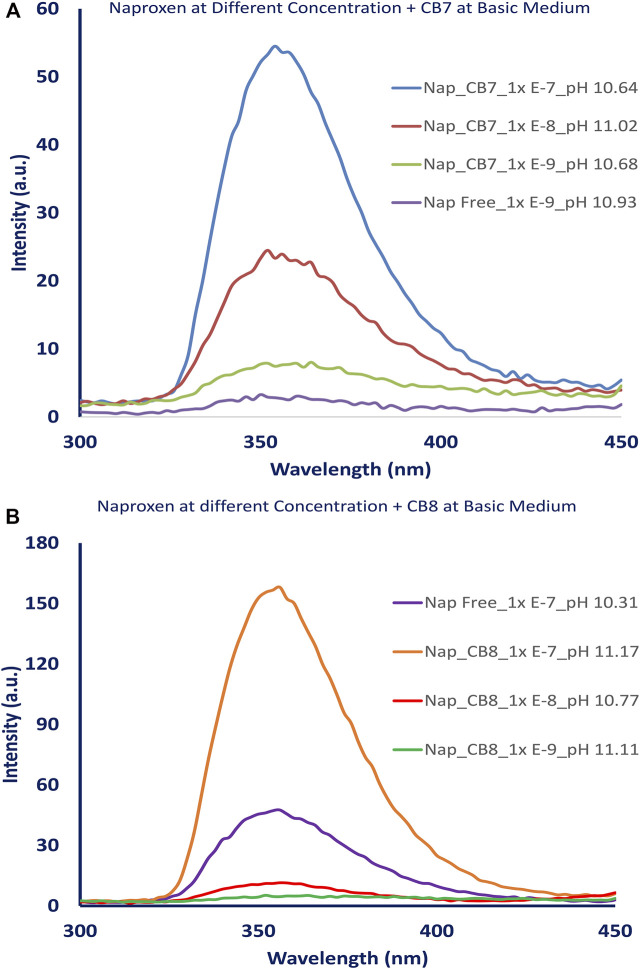
Fluorescence spectra of naproxen at different concentrations (1.00 x 10^−7^ − 1.00 x10^-9^ M) **(A)** without and with CB7, [CB7] = 0.176 mM., **(B)** without and with CB8, [CB8] = 0.176 mM.

The fluorescence intensity of the naproxen increased when CBs are added to the solution. Figure 2A shows the fluorescence of naproxen at various pH conditions without CBs; a slight increase in fluorescence intensity was observed as the pH changed from acidic to basic values. When CBs were added to the solution, the fluorescence intensity of naproxen increased. The addition of cucurbiturils to naproxen improved its fluorescence signal and allowed the detection of low concentrations as low as 10^−9^ M (see Figures 3A,B). While the addition of 0.01 equivalent of CB8 reduces the fluorescence intensity by tenfold, the addition of CB7 hosts causes CB7 a 20-fold increase in emission intensity (Figure 2B) indicating the establishment of a host-guest inclusion complex between CB7 and naproxen due to confinement effects. The emission is multiplied by four when CB8 up to one equivalent is added. (Figure 2C).

### 3.1 Formation of guest-host supramolecular complex

**FIGURE 4:**
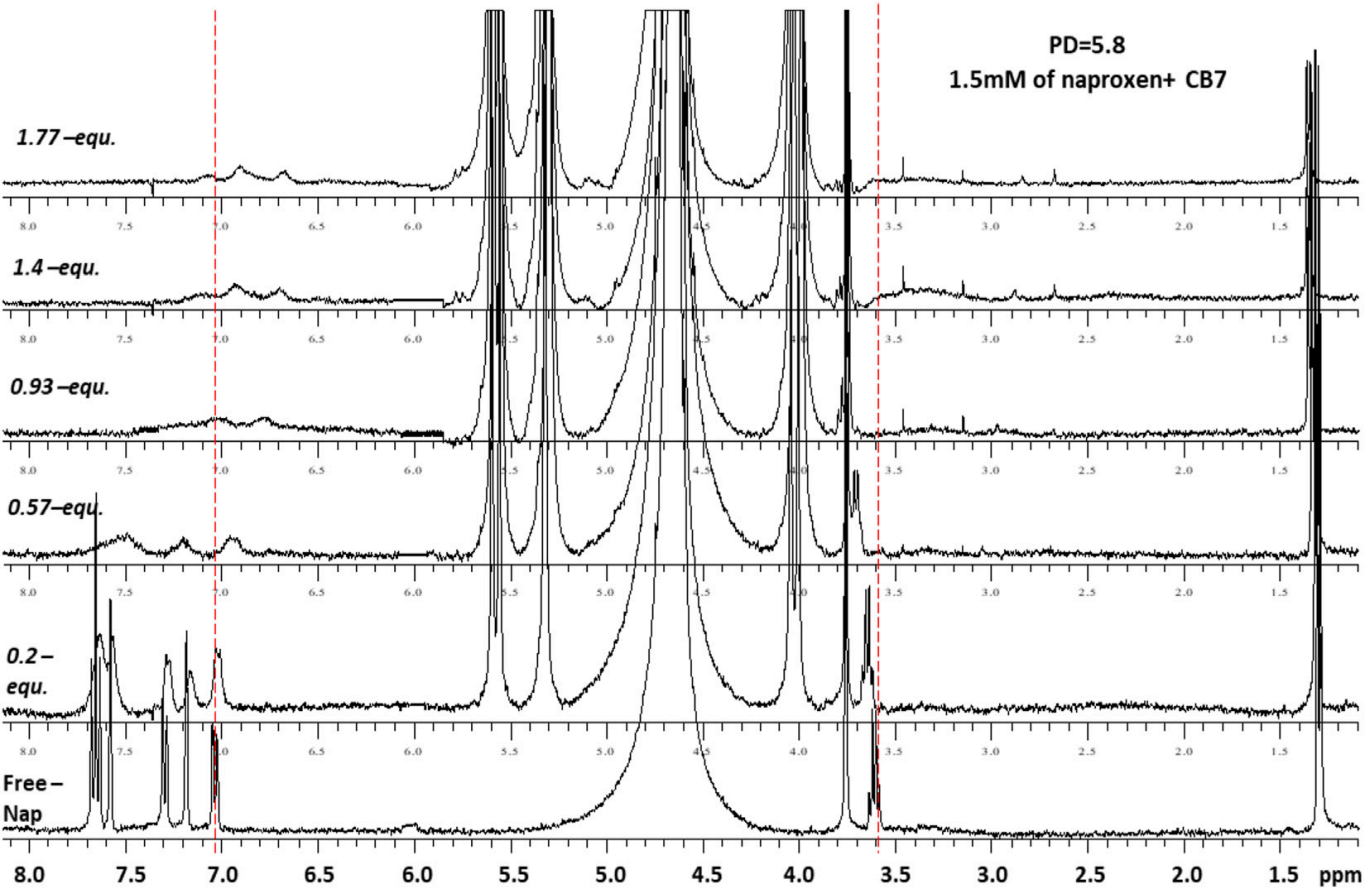
^1^H-NMR spectra (400 MHz, D2O) of naproxen (1.5 mM) in the absence (free) and presence pf 0-1.8 equivalents of CB7 at 298 K and pD 6.

**FIGURE 5:**
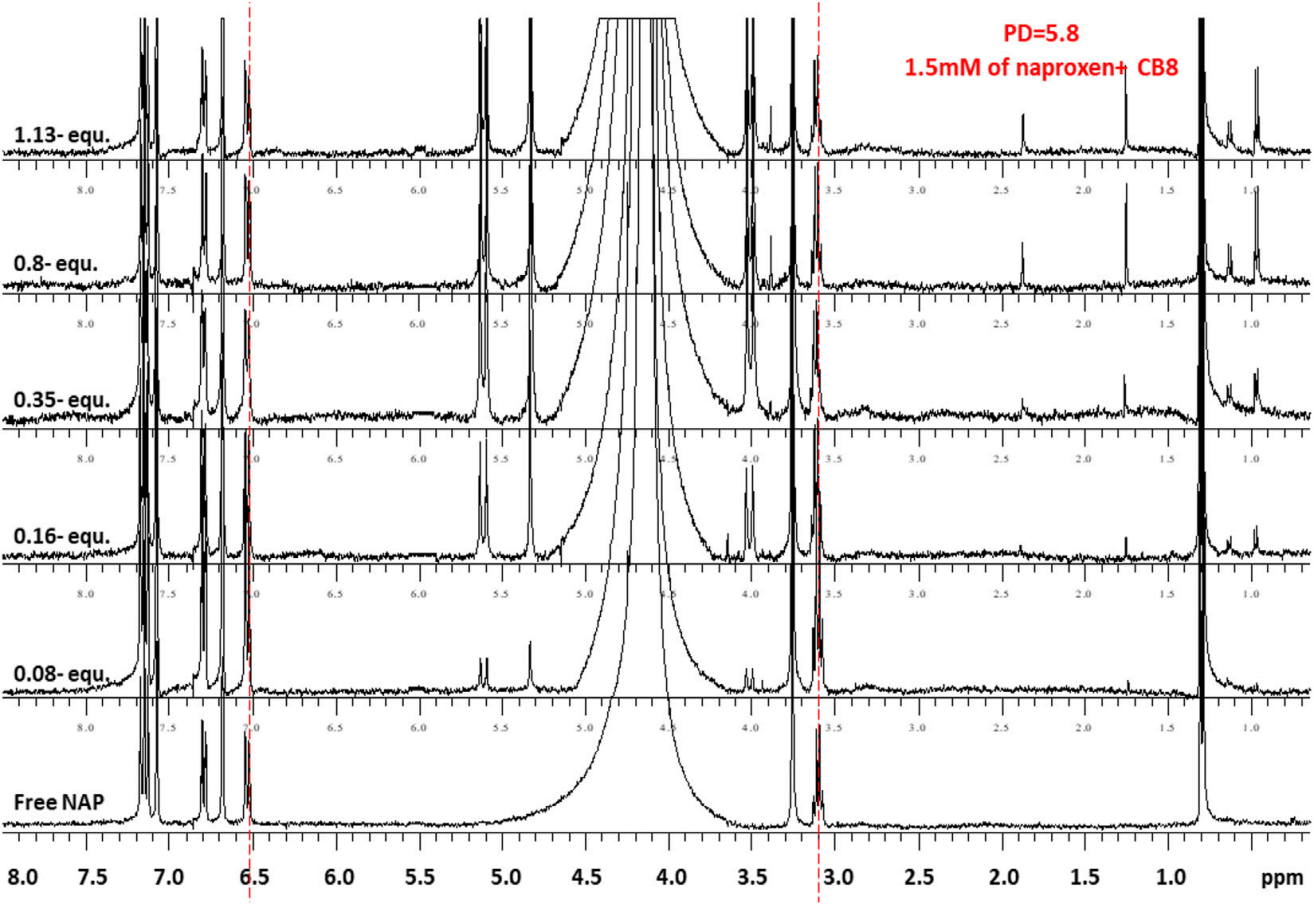
^1^H-NMR spectra (400 MHz, D_2_O) of naproxen (1.5 mM) in the absence (free) and presence pf 0-1.1 equivalents of CB8 at 298 K and pD 6.

**FIGURE 6:**
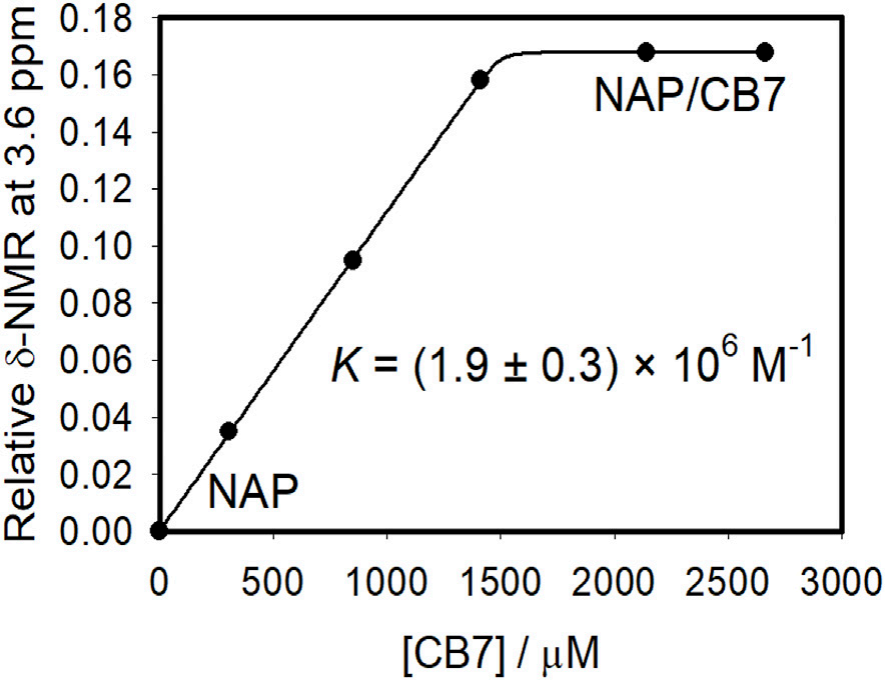
Binding curve resulted from the NMR spectral measurements of naproxen (1.5 mM) with different concentrations of CB7 at pH 6 in aqueous solution with the nonlinear fit to a 1:1 host−guest binding model (solid line and Experimental Section). The peak at ∼3.59 ppm (CH) was monitored to construct the binding titration plot. Δδ represents the difference between the NMR peak for CH in the absence and presence of CB7 (b).

**FIGURE 7:**
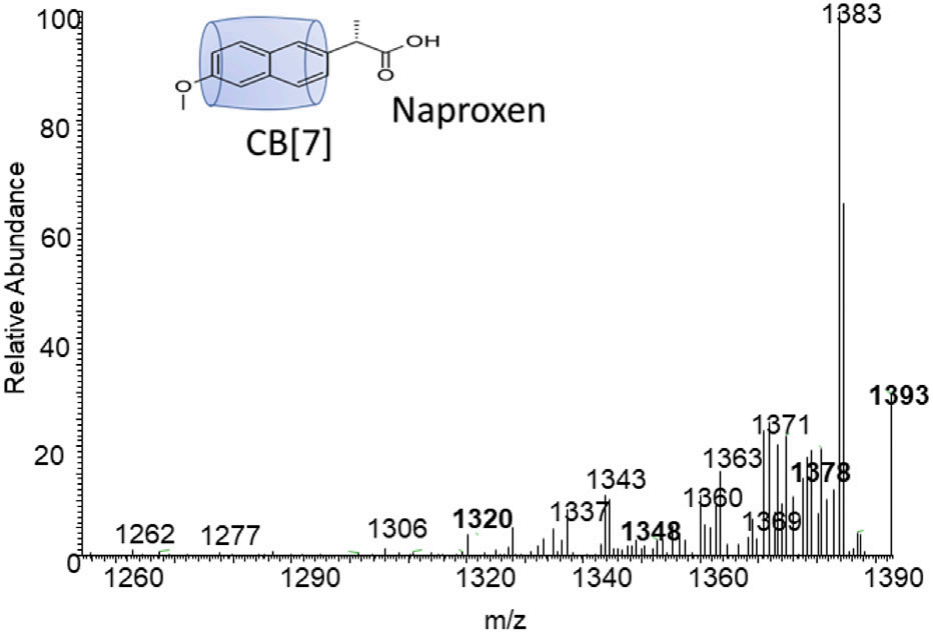
Electrospray tandem mass spectrometry of m/z 1393 which represent the mass of the naproxen-CB7 complex.

Figures 4, 5 demonstrate the use of ^1^H-NMR spectroscopy to investigate the type of interaction, stoichiometry, and mode of inclusion at pH 6. When 1 molar equivalent of CB7 was added to a naproxen solution, aromatic proton resonances corresponding to naphthalene unit protons shifted to lower ppm values with approximately 0.4 ppm, indicating their encapsulation into the hydrophobic cavity of CB7, whereas two resonance protons of quartet CH and doublet CH_3_ units shifted to higher ppm values (from 3.6 ppm to 1.3 ppm–3.8 ppm and 1.4 ppm, respectively). The singlet -OCH_3_ proton resonance was unaffected. When one molar equivalent of CB8 was added to naproxen solution, only the proton peak at 1.3 was replaced by two protons at 1.1 and 0.9 ppm, while all other resonances remained unchanged. Details on all of the corresponding binding titrations using NMR spectra and the additions of more than one equivalent of CB7 and CB8 can be found in the experimental section. Moreover, by monitoring the shifts in the NMR peak position of the CH proton at 3.6, we were able to extract a 1:1 host-guest stability constant (Figure 6) of (1.9 ± 0.3) × 10^6^ M^−1^ at approximately pH 6 (PD 6). In the NMR data at pD 6, the shifts observed after the addition of CB8 revealed a low binding affinity Figure 7 shows electrospray tandem mass spectrometry measurements of aqueous mixtures of naproxen and CB7 at m/z 1394. The complex fragmentation was revealed by the product ion scan MS/MS measurement, which revealed the loss of a fragment with a mass of 73 Da, which represents the propanoic acid (-CH(CH_3_)-COOH). This could indicate the inclusion of the naphthyl group and–OCH_3_ inside the CB7 cavity as well as the allocation of the COOH group close to the CB7’s carbonyl groups *via* intermolecular interactions (see the NMR results).

### 3.2 Application: Detection of naproxen drug in wastewater

As an application for the naproxen - CB7 interaction, an HPLC method for detecting naproxen in wastewater was developed. Initially, external calibration curves for naproxen were built with and without the addition of CB7 as a modifier to the mobile phase. When CB7 was added to the mobile phase, the slope of the calibration curve was significantly improved - an increase of one order of magnitude was observed (data not shown).

**FIGURE 8:**
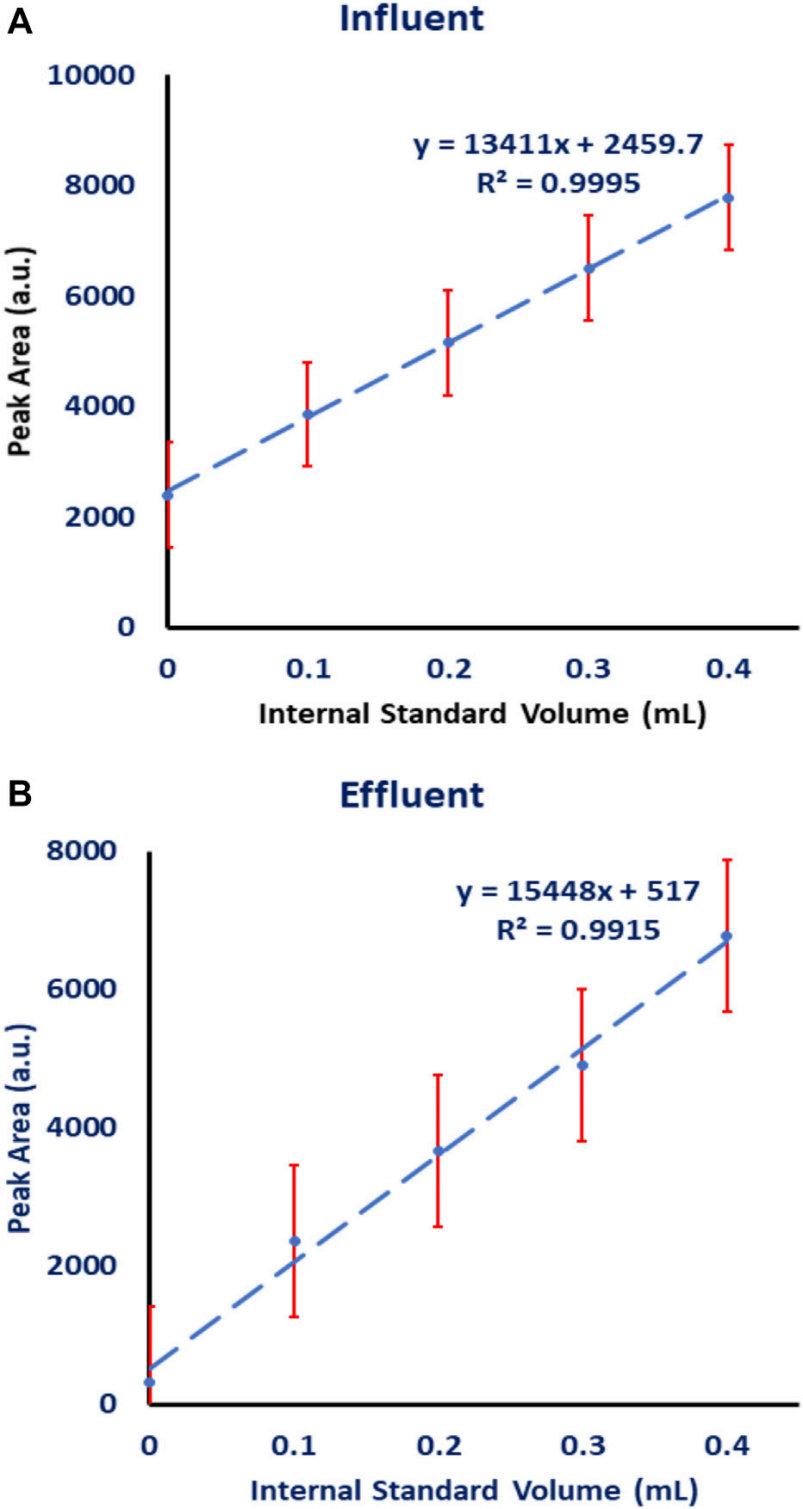
Standard addition calibration curve for naproxen analyzed on HPLC-FLD in the presence of CB7 added to the mobile phase. The unknown volume was 0.2 mL and the concentration of spiked naproxen standard was 100 ppb and its volume was 0.1 mL. **(A)** influent wastewater, **(B)** effluent wastewater.

Because wastewater is considered a complex mixture, detecting naproxen without interferences is unlikely. As a result, a standard addition calibration curve was investigated and used to estimate the levels of naproxen drug in wastewater influent and effluent samples collected from a wastewater treatment plant in Al-Ain, United Arab Emirates. Each sample of influent and effluent wastewater sample was extracted and concentrated separately, then collected in a 40 ml vial and evaporated until dry. The sample was reconstituted in a (85:15) (water: ethanol) solution and divided equally into five vials. To create a standard addition calibration curve, a standard naproxen solution with a concentration of 100 ppb was prepared and spiked into these five vials with varying volumes (0.0, 0.1, 0.2, 0.3, and 0.4 ml). Each vial was filled with water-ethanol solution to a total volume of 1.0 ml. The samples were then analyzed using HPLC-FLD. Figure 8 depicts the standard addition calibration curves for the influent and effluent samples. Each sample of wastewater was measured three times.

The extracted influent wastewater contained 0.187 ± 0.012 ppb, while the extracted effluent wastewater contained 0.021 ± 0.013 ppb. The actual concentration of naproxen in the influent wastewater is 1.87 × 10^−4^ ppb and 2.1 × 10^−5^ ppb in the effluent because samples were concentrated from 1.0 L (i.e., the analyzed sample was enriched 1000 times).

## 4 Discussion

Early reports show naproxen detection and quantification in tablets and human plasma using spectrofluorometric determination in the range of 0.2–2.0 mg/L (Damiani et al., 2002; Hammad et al., 2021), which agrees with our results (10^−8^ M, which is equivalent to 2.3 µg. L^−1^). The addition of CB7, on the other hand, improved the detection of lower concentrations of naproxen and lowered the detection limit to 0.23 µg. L^−1^ (10^−9^ M) due to the formation of a host-guest inclusion complex by confinement effects between CB7 and naproxen.

The UV-Vis and fluorescence measurements revealed an interaction between the naproxen drug and the CB host. Because naproxen has a pKa value of 4.2 that is associated with the carboxylic group, the intensity of naproxen fluorescence changed noticeably when the pH was changed from acidic to neutral to basic conditions. For example, at pH 6–10 most of this drug will be charged, having a carboxylate group rather than the carboxylic unit. However, the fact that fluorescence enhancement for naproxen was observed at different pH conditions in the presence of CB7 or CB8 could be explained by the hydrophobic part of the compound preferring the hydrophobic microenvironment inside the CB7 or CB8 cavity. Specifically, the macrocycle CB7 or CB8 prefers cationic or neutral moieties over anionic moieties, which also explains the mode of inclusion determined by the NMR and MS experiments below.

The addition of CB7 or CB8 to naproxen improved its fluorescence signal and enabled the detection of low concentrations at 10^−9^ M as shown in Figures 3A,B. However, the addition of 0.01 equivalent of CB8 causes the emission intensity to decrease by tenfold, while the addition of CB7 caused a 20-fold increase in naproxen emission intensity (Figure 2B) due to the formation of a host-guest inclusion complex between CB7 and naproxen as a result of confinement effects. The addition of CB8 up to 1 equivalent increases the emission by a factor of four (Figure 2C). This observation could be attributed to the difference in CB7’s internal cavity diameters (0.73 nm) and CB8 (0.88 nm) (Kim et al., 2007).

The naproxen compound is fitting properly the internal cavity of CB7, as indicated by the formation constant, (1.9 ± 0.3) × 10^6^ M^−1^; while the fitting of naproen cavity of CB8 is loose as indicated by almost no changes during the NMR titration. The NMR data established the encapsulation of naphthalene and O-CH_3_ units inside the CB7 cavity while the tandem MS result confirmed the position of the propanoic acid (-CH(CH_3_)-COOH) group outside the CB7 cavity, since it was easily fragmented during the MS/MS experiment.

Several studies have been published in peer-reviewed journals that describe the detection of naproxen in wastewater using HPLC with UV-Vis, fluorescence, or mass spectrometry (Rabiet et al., 2006; Matamoros et al., 2009; Payán et al., 2011; Madrakian et al., 2013; Asadi et al., 2015; Durán-Álvarez et al., 2015; Madikizela & Chimuka, 2017a; Madikizela & Chimuka, 2017b; García-Herrero et al., 2019; Khazri et al., 2019; Ma et al., 2019; Mandaric et al., 2019; Dionísio et al., 2020; Maraqa et al., 2020). In the preconcentration and enrichment step, various extraction and concentration techniques were employed, such as solid phase extraction (Maraqa et al., 2020), electro-membrane extraction (Payán et al., 2011), and Vortex-assisted surfactant-enhanced emulsification microextraction based on solidification of floating organic droplets (Asadi et al., 2015). These extraction methods made it possible to accurately identify naproxen in wastewater samples at concentrations as low as ppb. For example, Madikizela & Chimuka. (2017b) used solid phase extraction (SPE) with HPLC photo diode array detection to quantify naproxen and two other drugs in wastewater. The pH of wastewater samples was adjusted to pH 2.5 before loading 100 ml of each sample onto a pre-conditioned cartridge for SPE. They discovered naproxen levels in influent and effluent wastewater were in the 15–20 μg/L and 0.6–1.1 μg/L ranges, respectively. In another study, an electro-membrane extraction (EME) combined with an HPLC procedure using diode array (DAD) and fluorescence detection (FLD) for the determination of naproxen and other nonsteroidal anti-inflammatory drugs in another study. The method was applied successfully to urban wastewaters with detection and quantitation limits of 0.0009–9.0 and 0.003–11.1 μg. L^−1^, respectively (Payán et al., 2011).

These findings are comparable to those obtained in our current study. The extracted influent wastewater had an average naproxen concentration of 0.187 ± 0.012 ppb, while the extracted effluent wastewater had a concentration of 0.021 ± 0.013 ppb. The actual concentration of naproxen in the influent wastewater is 1.87 × 10^−4^ ppb and 2.1 × 10^−5^ ppb in the effluent because these concentrations were reported after sample enrichment from 1000 ml to 1.0 ml.

## 5 Conclusion

We used a guest-host interaction in this study to show how the CB7 and CB8 microenvironments affect the fluorescence behavior of the naproxen drug. The supramolecular interactions between the drug (guest) and CB7 (host) are linked to the host stiffness and electrostatic forces. The study used naproxen signal enhancement in the presence of CB7, which demonstrated its unique hosting properties of high binding constant to naproxen drug, to develop a sensitive method of detecting the naproxen drug in complex wastewater matrices. Solid phase extraction was employed to abstract and concentrate the drug from the wastewater samples. HPLC-FLD analysis in the presence of CB7 as an additive in the mobile phase was used for selective chromatographic separation and sensitive fluorescence detection of naproxen drug in wastewater samples. The naproxen levels in the influent and effluent wastewater were 1.87 × 10^−4^ ppb and 2.1 × 10^−5^ ppb, respectively.

## Data Availability

The original contributions presented in the study are included in the article/Supplementary Material, further inquiries can be directed to the corresponding author.
